# Prospective evaluation of clinical symptoms of chemotherapy‐induced oral mucositis in adult patients with acute leukemia: A preliminary study

**DOI:** 10.1002/cre2.253

**Published:** 2019-12-09

**Authors:** Yeon‐Hee Lee, Junshik Hong, Inho Kim, Youngnim Choi, Hee‐Kyung Park

**Affiliations:** ^1^ Department of Orofacial Pain and Oral Medicine Kyung Hee University Dental Hospital Seoul Korea; ^2^ Department of Internal Medicine Seoul National University Hospital, Seoul National University College of Medicine Seoul Korea; ^3^ Cancer Research Institute Seoul National University Seoul Korea; ^4^ Department of Immunology and Molecular Microbiology, School of Dentistry and Dental Research Institute Seoul National University Seoul Korea; ^5^ Department of Oral Medicine and Oral Diagnosis, Dental Research Institute Seoul National University Dental Hospital, Seoul National University School of Dentistry Seoul Korea

**Keywords:** adults, chemotherapy‐induced oral mucositis (CIOM), hematopoietic stem cell transplantation (HSCT), leukemia, WHO score

## Abstract

**Objective:**

The objective of this study was to prospectively evaluate the clinical features of chemotherapy‐induced oral mucositis (CIOM) in adult patients with acute leukemia and the aggravating factors for such symptoms.

**Subjects:**

Thirty‐seven prospective patients aged ≥19 years with acute leukemia undergoing chemotherapy were enrolled. Oral and clinical investigations were performed at baseline and on Day 14 after starting chemotherapy. The presence and severity of cancer‐induced oral mucositis were demonstrated using the World Health Organization (WHO) scoring system.

**Results:**

On Day 14, we found that oral mucositis had developed in eight patients (21.6%). Hematopoietic stem cell transplantation (HSCT) in patients was a predictor of increased WHO scores (*β* = 1.937, *p* < .001). Regarding oral sites, ventral tongue (*β* = 1.670), soft palate (*β* = 1.242), and buccal mucosa (*β* = 0.593) were predictors for increased scores. In addition, the increase in WHO scores was positively correlated with the number of oral lesions (*r* = .521), the difficulty in eating (*r* = .250), and the overall oral health (*r* = .534; all *p* < .05).

**Conclusion:**

The main factors affecting the severity of CIOM symptoms were the treatment with HSCT and the location of oral lesions. The incidence of CIOM and WHO scores were not significantly different between the subgroups of disease. Our findings will help clinicians investigate the oral findings after chemotherapy in adult patients with acute leukemia.

## 1 INTRODUCTION

Leukemia is a kind of hematological cancer that is caused by an overproduction of white blood cell (WBC)‐forming tissues resulting in a marked increase in circulating immature or abnormal WBCs (Shysh et al., 2017). Leukemia is an uncommon disease, and the crude incidence rate per 100,000 was 6.4 in a Korean population (Jung, Won, Kong, Lee, & The Community of Population‐Based Regional Cancer, 2018). As the number of abnormal WBCs in acute leukemia increases faster than that in chronic leukemia, acute leukemia can be more deleterious (Belson, Kingsley, & Holmes, 2007). The 2016 revision to the World Health Organization (WHO) classification of acute leukemia includes two main subtypes, namely, acute lymphoid leukemia (ALL) and acute myeloid leukemia (AML), according to their prognostic, morphological, immunophenotypic, genetic, and clinical traits (Arber et al., 2016). AML is the commonest type of acute leukemia in adults (Yamamoto & Goodman, 2008), whereas ALL occurs more frequently than AML in childhood and rarely occurs in adults (Belson, Kingsley, & Holmes, 2007; Gurney, Severson, Davis, & Robison, 1995; Shysh et al., 2017). Thus, oral mucositis in adult patients with AML has not yet been fully assessed.

Chemotherapy involves the use of chemicals or drugs that destroy or prevent the reproduction of cancer cells in the leukemia patients. Approximately 80% of newly diagnosed patients with AML are treated with cytarabine, anthracyclines, such as daunorubicin or idarubicin, and cladribine (Buchner et al., 2003; Ohno et al., 1993; Rashidi, Walter, Tallman, Appelbaum, & DiPersio, 2016). Clinically, patients under the age of 60 often receive intensive chemotherapy, and induction often involves treatment with two or more of the chemotherapeutic drugs. The adverse effects of chemotherapy are related to their cytotoxic activities against noncancerous cells of the body, resulting in undesirable anatomical and functional conditions such as dysphagia, vomiting, diarrhea, malnutrition, arthralgia, hemorrhage, anemia, and myelosuppression (Sonis et al., 2004).

The oral cavity is also affected as a result of local and/or systemic side effects of chemotherapy, whereby oral lesions are called chemotherapy‐induced oral mucositis (CIOM; Chen et al., 2011). Oral mucositis is inflammation of the oral mucosa, occurring in 20–40% of patients receiving conventional chemotherapy (Lionel, Christophe, Marc, & Jean‐Luc, 2006; Naidu et al., 2004; Scully, Sonis, & Diz, 2006). Chemotherapy causes inflammation and ulceration through tissue damage resulting from a sequence of chemical, metabolic, and biological events that occur in several stages (Lopez‐Castano, Onate‐Sanchez, Roldan‐Chicano, & Cabrerizo‐Merino, 2005). Thus, CIOM usually begins within the first week after the initiation of treatment and peaks in the second week (Lionel et al., 2006; Naidu et al., 2004; Scully et al., 2006). Oral health plays an important role in the quality and life expectancy of an individual. However, the incidence and clinical characteristics of CIOM in patients with acute leukemia are still lacking.

Some patients with acute leukemia may receive allogeneic hematopoietic stem cell transplantation (HSCT), which involves profound immunosuppression and various related complications. The incidence of oral mucositis among patients receiving HSCT is reportedly up to 70% (Vagliano et al., 2011), which is much higher than in patients who receive conventional chemotherapy. Oral mucosal disruption caused by CIOM can be fatal to patients because it provides a major route for the entry of pathological microorganisms, leading to various infections including fatal septicemia (Ruescher, Sodeifi, Scrivani, Kaban, & Sonis, 1998). A significant percentage of oral lesions that are caused by anticancer treatments may be reversible, whereas little is known about how HSCT affects CIOM in patients with acute leukemia.

The aim of the current study was to prospectively determine the clinical features of CIOM in adult patients with acute leukemia and to compare their oral signs and symptoms between the two subgroups, ALL and AML. Additionally, we also aimed to determine the risk factors that aggravate the severity of CIOM. That is, we evaluated whether clinical factors such as location of the oral lesion, the number of lesions, subtype of acute leukemia, and treatment with HSCT affect the development and severity of CIOM in patients with acute leukemia. The understanding of specific oral manifestations in adult patients with leukemia following chemotherapy can facilitate the perdition of novel treatment and result in the ability to correlate causative factors to the severity of CIOM.

## 2 MATERIALS AND METHODS

### 2.1 Patients and study design

We performed a prospective, observational clinical study on adult population of patients with acute leukemia, comprising both ALL and AML forms. The diagnosis of acute leukemia subtypes ALL and AML was based on the 2016 WHO criteria (Barbui et al., 2018). Medical records of each participant were reviewed at baseline, and their demographic and clinical characteristics such as age, sex, subtype of acute leukemia, and treatment with HSCT were investigated.

The inclusion criteria for the study were as follows: (a) age ≥ 19 years, (b) diagnosed with an acute leukemia and admitted for intensive chemotherapy, (c) did not have any oral lesions at baseline, and (d) had not undergone or recovered from prior chemotherapy, radiation therapy, or surgery prior to enrollment. The exclusion criteria were as follows: (a) patients with other types of leukemia, (b) patients who already had definitive symptoms or signs of oral mucositis at baseline, (c) those who had other severe dental and/or systemic diseases, and (d) those who had underlying psychological diseases or cognitive disorders that precluded necessary communication. All enrolled patients commonly received intensive chemotherapy and, in some cases, with allogeneic HSCT.

The patients were evaluated twice (at baseline and Week 2) during each enrollment. At the initiation of chemotherapy (baseline examinations), all patients received constructive oral examinations to confirm that no oral lesions were present. At Week 2 of enrollment, a series of clinical and hematological tests were performed on patients to identify symptoms objectively, as well as subjectively. In the present study, we analyzed total WBC, lymphocyte and absolute lymphocyte counts (ALC), absolute neutrophil count, and C‐reactive protein (CRP) as hematological factors.

Standard infection prevention measures that were applied included isolation with a high efficiency particulate air‐filtered laminar flow hood, low‐bacteria diet during the neutrophil count nadir, and hand hygiene practices together with the use of surgical masks during patient contact.

This prospective cohort study was performed in accordance with the principles in the 1964 Declaration of Helsinki and its later updated version, and our study was approved by the Institutional Review Board of Seoul National University School of Dentistry, Seoul, Korea (approval number: S‐D20160016). Written informed consent was obtained from all patients as a condition of participation.

### 2.2 Intensive chemotherapy regimens

Patients with AML or ALL were given standard intensive chemotherapy for curative intent after clinical judgement that they would be able to tolerate high‐dose chemotherapy. For patients with AML, conventional 3 + 7 intensive chemotherapy (12 mg·m^−2^·day^−1^ of idarubicin or 60–90 mg·m^−2^·day^−1^ of daunorubicin for three consecutive days plus 100 mg·m^−2^·day^−1^ of continuous cytarabine infusion for 7 days) was conducted for remission induction and either high‐dose (3 g·m^−2^·day^−1^ for Days 1, 3, and 5) or intermediate‐dose (2 g·m^−2^·day^−1^ for Days 1, 3, and 5) cytarabine was administered as consolidative chemotherapy. Patients with ALL received multiagent combination chemotherapy, according to either vincrisintine, prednisolone, daunorubicin, and L‐asparaginase protocol (Park et al., 2003) or hyper‐cyclophosphamide, vincristine, adriamycin, and dexamethasone alternating with high‐dose methotrexate and cytarabine regimen (Thomas et al., 2004). All ALL patients received appropriate central nervous system prophylaxis using intrathecal chemotherapy with methotrexate with or without cytarabine and hydrocortisone. ALL patients who had positive result for the breakpoint cluster region–proto–oncogene tyrosine–protein kinase rearrangement according to fluorescence in situ hybridization or polymerase chain reaction test, 600 mg·day^−1^ of imatinib mesylate was added on the multiagent chemotherapy.

### 2.3 Measurement of CIOM

At baseline, the authors divided the oral cavity into eight regions and investigated the presence of CIOM: buccal mucosa, dorsal surface of the tongue, ventral surface of the tongue, the gingiva, floor of the mouth, hard palate, soft palate and tonsil, and labial mucosa.

The clinical features, including the presence or absence of CIOM, the location, and the severity of the lesion, were investigated. At Week 2, the presence and severity of CIOM presence and severity were estimated and graded according to the WHO's oral toxicity scale. The WHO scale combines the subjective and objective measures of oral mucositis as follows: Grade 0 = No oral mucositis, Grade 1 = Erythema and soreness, Grade 2 = Ulcers, able to eat solids, Grade 3 = Ulcers, requires liquid diet due to mucositis, and Grade 4 = Ulcers, alimentation not possible due to mucositis (Lalla, Sonis, & Peterson, 2008). The WHO score ranges from 0 to 4 points (Grades 0 to 4), with higher scores indicating greater severity of the CIOM status. This scale is a simple and easy‐to‐use measure that is appropriate for clinical applications. For patients with more than one lesion in the mouth, the most severe site was investigated. Two clinicians with at least 10 years of experience in the field, and who were blinded to the patients' medical information, performed the oral examination and visual analyses of the CIOM. This scale has been validated with high‐interobserver reproducibility.

### 2.4 Subjective discomfort in patients

On Day 14, all patients reported their subjective discomfort using the oral mucositis daily questionnaire (OMDQ; Stiff et al., 2006). This questionnaire was designed to score the degree of discomfort felt by the patient during the preceding 24 hr. Of the total of 10 questions, two questions related to diarrhea were excluded leaving only eight questions to be answered by the patients in the present study.

The OMDQ sought information on the degree of subjective discomfort for the following two factors on a scale of 0 to 10: (a) overall health and (b) overall mouth and throat soreness. Other factors related to daily activities rated on a scale of 0 to 4 were as follows: (a) mouth and throat soreness, (b) swallowing, (c) drinking, (d) eating, (e) talking, and (f) sleeping. For all questions in the OMDQ, a higher score indicated worsening symptoms or more interference with functional activities.

### 2.5 Statistical analysis

We obtained the absolute and percentage distributions of all nominal and categorical variables, as well as means and standard deviations, and performed descriptive data analysis. Results of ALL and AML patients were compared using the Mann–Whitney *U* test. The Fisher's exact test was used to determine equality of the proportions. Estimated beta (β) was estimated with linear regression models. In the linear regression model, the dependent variable was the WHO score, and the other factors were considered the explanatory variables. Spearman's correlation analyses were performed to examine the correlations among WHO score, number of areas, and the various subjective discomforts. All statistical analyses were calculated using IBM SPSS Statistics for Windows, Version 20.0 (IBM Corp., Armonk, NY, USA). Statistical significance was established at *p* values <.05.

## 3 RESULTS

### 3.1 Patient enrollment and treatment

**Figure 1:**
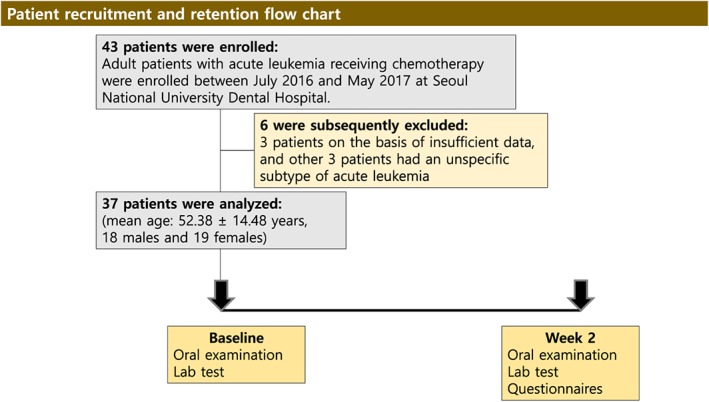
Patient recruitment and retention flow chart

Patients were enrolled between July 2016 and May 2017 at Seoul National University Dental Hospital. Initially, 43 participants with acute leukemia were recruited. However, six were subsequently excluded, three patients on the basis of insufficient data, and three other patients because they had an unspecific subtype of acute leukemia (Figure 1). Thus, 37 adult patients (52.38 ± 14.48 years, 18 males and 19 females) were finally included and evaluated. Of this cohort of patients, 32 had AML (53.56 ± 13.82 years) and five had ALL (44.80 ± 17.98 years). Overall, the male‐to‐female ratio of adult patients with acute leukemia was 0.95:1, and those of the various subtypes were 1.33:1 and 0.25:1 for AML and ALL, respectively. Overall, the male‐to‐female ratio is similar to that of the whole Korean population, which has been reported as 1.33:1 (Jung et al., 2018).

Our results for adults aged 19 years or older show that the number of AML patients is 6.4 times more than that of ALL patients. It has been reported that the incidence of AML increases with age, from ~1.3 cases per 100,000 population in patients less than 65 years old to 12.2 cases per 100,000 population in those over 65 years (Siegel, Miller, & Jemal, 2015). Clinical demographics and characteristics of the enrolled patients are summarized in Table 1.

**Table 1:** Demographics and results of lab test

Parameter	Leukemia (*n* = 37)	AML (*n* = 32)	ALL (*n* = 5)	*p* value
Sex ratio				
Male, *n* (%)	18 (48.6)	17 (53.1)	1 (20.0)	.187
Female, *n* (%)	19 (51.4)	15 (46.9)	4 (80.0)	
Age	52.38 ± 14.48	53.56 ± 13.82	44.80 ± 17.98	.213
Lab test				
WBC (/μl)	1,136.67 ± 3,480.48	1,318.21 ± 3,759.14	120.00 ± 43.01	.103
Lymphocytes (%)	76.29 ± 8.98	**73.23 ± 9.54**	**92.20 ± 4.55**	**.017** *
ALC (/μl)	290.42 ± 459.29	**322.29 ± 492.76**	**112.00 ± 40.85**	**.035** *
ANC (/μl)	577.97 ± 2,054.04	680.75 ± 2,219.93	2.40 ± 5.37	.118
CRP (mg·L^−1^)	4.89 ± 7.49	**5.57 ± 7.95**	**1.08 ± 1.31**	**.009** **

*Note.* Results were obtained via Mann–Whitney *U* test and chi‐squared test; *p* value significance was set at <.05. Significant variables showed in bold text. Standard range: WBC: 4,000–10,000/μl; lymphocytes: 15–52%; ALC: 1.0–5.1/μl; ANC: 2.0–8.0/μl; and CRP: 0–10 mg·L^−1^.

Abbreviations: ALC, absolute lymphocyte count; ALL, acute lymphoid leukemia; AML, acute myeloid leukemia; ANC, absolute neutrophil count; CRP, C‐reactive protein; WBC, white blood cell.

* *p* value <.05.

** *p* value <.01.

### 3.2 Hematological factors

Table 1 also shows the mean and standard deviation of hematological factors. Lymphocyte level was significantly lower in AML, whereas ALC and CRP levels were significantly higher in AML (all *p* < .05). Other hematological factors showed no significant differences. The reference normal ranges of each variable are as follows: WBC: 4,000–10,000/μl, lymphocytes: 15–52%, ALC: 1.0–5.1/μl, absolute neutrophil count: 2.0–8.0/μl, and CRP: 0–10 mg·L^−1^.

### 3.3 Incidence, location, and the severity of CIOM

**Figure 2:**
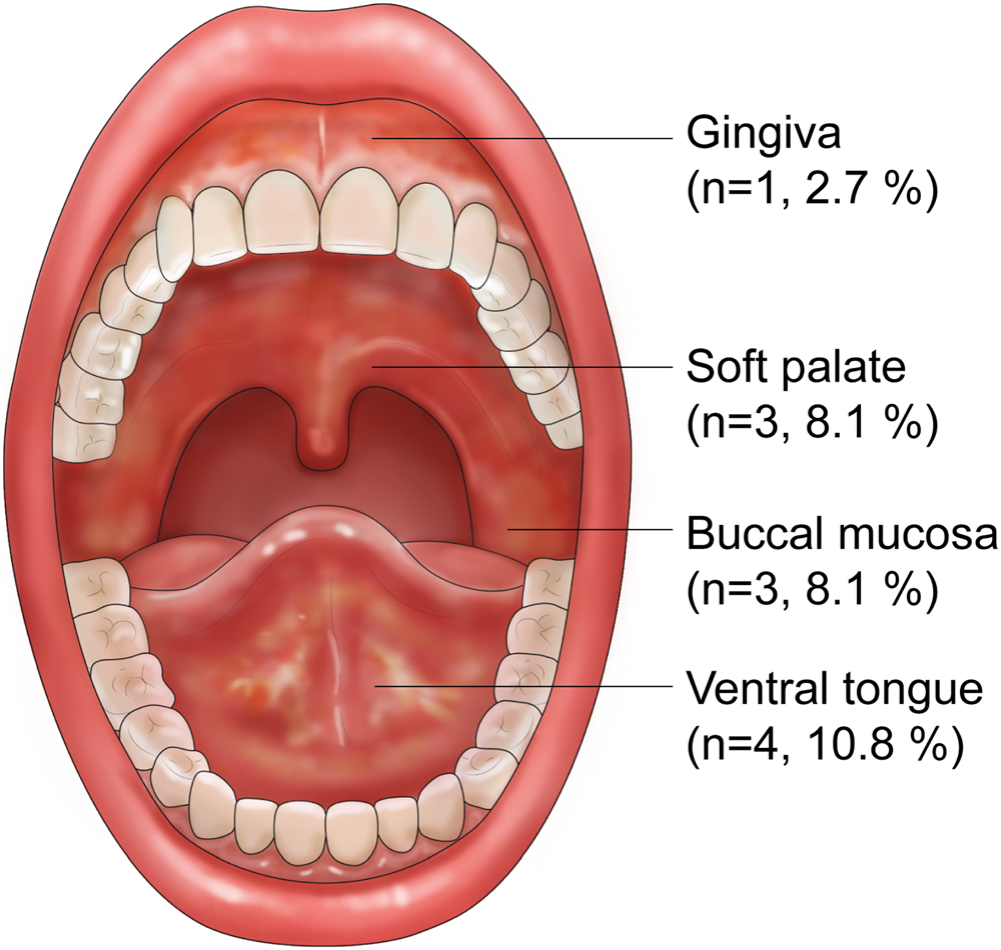
Distribution of chemotherapy‐induced oral mucositis in patients with acute leukemia

Figure 2 shows the distributions of CIOM, and Table 2 presents the differences according to the subtype of acute leukemia. Regarding the overall incidence of CIOM in 37 patients with acute leukemia undergoing intensive chemotherapy, eight of 37 (21.6%) patients with acute leukemia experienced CIOM at Week 2. Based on the WHO oral mucositis grading scale, three out of eight patients were classified as WHO Grade 1 with simple erythema in the oral cavity, and five patients were classified as Grade 2 with ulcer. None was in either Grade 3 or Grade 4. In general, CIOM is typically less severe and usually lasts for less than 2 weeks (Georgiou, Patapatiou, Domoxoudis, Pistevou‐Gompaki, & Papanikolaou, 2012). The incidence of CIOM was found to be 21.9% in the AML, and 20.0% in the ALL group. The numbers of CIOM, their distribution in the oral cavity, and the severity of CIOM were not significantly different between the two subgroups.

**Table 2:** Comparison of lesion's distributions between groups

Parameter	Leukemia (*n* = 37)	AML (*n* = 32)	ALL (*n* = 5)	*p* value
Number of oral lesion (mean ± *SD*)	0.30 ± 0.66	0.31 ± 0.69	0.20 ± 0.45	.645
Presence of oral lesion, *n* (%)				
(1) Single lesion	6 (16.2)	5 (15.6)	1 (20.0)	.763
(2) Multiple lesions	2 (5.4)	2 (6.3)	0 (0.0)	
(1) + (2) total	8 (21.6)	7 (21.9)	1 (20.0)	1.000
Location, *n* (%)				
Buccal mucosa	4 (10.8)	4 (12.5)	0 (0.0)	.683
Ventral tongue	4 (10.8)	4 (12.5)	0 (0.0)	.544
Soft palate	3 (8.1)	3 (9.4)	0 (0.0)	.683
Gingiva	1 (2.7)	0 (0.0)	1 (20.0)	.135
WHO scores (mean ± *SD*)	0.35 ± 0.72	0.34 ± 0.70	0.40 ± 8.89	.865
WHO oral mucositis grading scale, *n* (%)^a^				
WHO Grade 0 (none)	29 (78.4)	25 (78.1)	4 (80.0)	.722
WHO Grade 1 (mild)	3 (8.1)	3 (9.4)	0 (0.0)	
WHO Grade 2 (moderate)	5 (13.5)	4 (12.5)	1 (20.0)	

*Note.* Results were obtained via Mann–Whitney *U* test and chi‐squared test; *p* value significance was set at <.05.

Abbreviations: ALL, acute lymphoid leukemia; AML, acute myeloid leukemia; WHO, World Health Organization.

a There were no patients with WHO Grades 3 (severe) and 4 (life threatening) in the present study.

Among the 32 AML patients, six (18.0%) patients received HSCT, and CIOM was observed in half of them. The commonest site of CIOM was the ventral tongue (*n* = 4, 10.8%), followed by the buccal mucosa (*n* = 3, 8.1%), soft palate (*n* = 3, 8.1%), and gingiva (*n* = 1, 2.7%). When the data were divided according to the subtype, only one case in which the gingiva was affected was observed in ALL patients. Among the 32 AML patients, CIOM was observed on the ventral tongue (*n* = 4, 12.5%), followed by the buccal mucosa (*n* = 3, 9.4%) and soft palate (*n* = 3, 9.4%). CIOM was not observed in other parts of the mouth with the exception of the aforementioned four sites. The severity of CIOMs was estimated according to the WHO scoring system, and it was not significantly different between the AML and ALL groups.

### 3.4 Subjective complaints of the patients

Table 3 shows the subjective complaints of oral pain or limitation of function using the OMDQ. When data were analyzed for the whole cohort of acute leukemia patients, a noticeably higher score was found for overall health status (4.97 ± 3.57) compared with that for overall mouth and throat soreness (1.88 ± 3.11). Upon investigating the degree of discomfort in daily activity, the highest value was found when eating (0.72 ± 1.34), followed by drinking (0.48 ± 1.16), swallowing (0.48 ± 1.01), sleeping (0.48 ± 0.92), and talking (0.24 ± 0.66).

**Table 3:** Results from the oral mucositis daily questionnaire

Parameter	Leukemia (*n* = 37)	AML (*n* = 32)	ALL (*n* = 5)	*p* value
Questionnaire (mean ± *SD*)				
Q1 (0–10), overall health	4.97 ± 3.57	5.22 ± 3.67	3.60 ± 2.88	.308
Q2 (0–4), mouth and throat soreness	0.56 ± 1.04	0.60 ± 1.14	0.40 ± 0.55	.581
Q3 (0–4), swallowing	0.48 ± 1.01	**0.60 ± 1.10**	**0.00 ± 0.00**	**.024** *
Q3 (0–4), drinking	0.48 ± 1.16	**0.60 ± 1.27**	**0.00 ± 0.00**	**.049** *
Q3 (0–4), eating	0.72 ± 1.34	0.85 ± 1.46	0.20 ± 0.45	.104
Q3 (0–4), talking	0.24 ± 0.66	0.30 ± 0.73	0.00 ± 0.00	.083
Q3 (0–4), sleeping	0.48 ± 0.92	**0.60 ± 0.99**	**0.00 ± 0.00**	**.014** *
Q4 (0–10), overall mouth and throat soreness	1.88 ± 3.11	2.05 ± 3.33	1.20 ± 2.17	.504

*Note.* Results were obtained via Mann–Whitney *U* test; *p* value significance was set at <.05. Significant variables showed in bold text.

Abbreviations: ALL, acute lymphoid leukemia; AML, acute myeloid leukemia.

* *p* value <.05.

When the data were divided into two subtypes of disease, AML and ALL, the subjective discomfort of swallowing, drinking, and sleeping was significantly higher in patients with AML than in those with ALL (all *p* < .05). In both the AML and ALL groups, the score of overall health status was higher than that of overall mouth and throat soreness. Among the factors related to daily activities, difficulty in eating was the highest in both subgroups. However, the second highest score was different between the two subgroups; drinking was the second highest rate in AML, whereas none of the patients felt discomfort during daily activities such as swallowing, drinking, talking, and sleeping. However, some patients with ALL reported having difficulty eating.

### 3.5 Factors associated with severity of CIOM

Table 4 shows the results of linear regression analyses as to which factors affect the increase in the WHO score when the severity of CIOM is expressed as the WHO score. We considered the WHO score as the dependent variable and the other clinical factors as the explanatory variables. Notably, treatment with HSCT significantly increased the WHO score (*β* = 1.937, *p* < .01). Regarding the location of the CIOM lesion, the ventral tongue (*β* = 1.670, *p* < .001) had the greatest effect on the increase of the WHO score, followed by the soft palate (*β* = 1.242, *p* < .001) and buccal mucosa (*β* = 0.593, *p* < .01). Of the hematological factors, only the decrease in leukocyte count (*β* = −0.012, *p* < .05) significantly affected the increase in the WHO score. Gender, age, subtype of acute leukemia, and other hematological factors did not significantly impact the WHO score.

**Table 4:** Stepwise multiple regression analysis with WHO scores as a dependent variable

	WHO scores		
Parameters	Beta (*β*)	*t* statistic	*p* value
Female	−0.558	−0.655	.517
Age	0.002	0.617	.542
The presence of			
AML	−0.187	−0.219	.828
ALL	0.188	0.219	.828
HSCT	**1.937**	**2.258**	**.001** **
Location			
Buccal mucosa	**0.593**	**3.149**	**.004** **
Ventral tongue	**1.670**	**10.236**	**<.001** ***
Soft palate	**1.242**	**7.610**	**<.001** ***
Lab test			
WBC	−2.888 E‐06	−0.032	.975
Lymphocyte %	**−0.012**	**−2.083**	**.048** *
ALC	0.000	−0.799	.432
ANC	0.000	−1.046	.306
CRP	0.005	0.209	.836
*R* ^2^	.964		

*Note.* Results were obtained via linear regression analysis; *p* value significance was set at <.05. Significant variables showed in bold text.

Abbreviations: ALC, absolute lymphocyte count; ALL, acute lymphoid leukemia; AML, acute myeloid leukemia; ANC, absolute neutrophil count; CRP, C‐reactive protein; HSCT, hematopoietic stem cell transplantation; WBC, white blood cell; WHO, World Health Organization; *β*, standardized regression coefficient.

* *p* value <.05.

** *p* value <.01.

*** *p* value <.001.

### 3.6 Correlations with CIOM severity

Table 5 shows which factors correlate with CIOM severity. Note that the strongest positive correlation with the WHO score was the number of oral lesions (*r* = .858, *p* < .001). Regarding the subjective discomfort identified in the OMDQ, the increase in WHO score was not related to concerns about overall health, but there were significant positive correlations with overall oral discomfort (*r* = .544, *p* < .001) and oral soreness (*r* = .521, *p* < .001). The increase in WHO score was positively correlated only with the increased discomfort of eating (*r* = .250, *p* < .05) among daily activities, and not with other activities including speaking or sleeping. The discomfort of daily activities including swallowing, drinking, eating, speaking, and sleeping was positively correlated with each other (all *p* < .05), but we observed that overall health and overall oral health were not significantly related to each other.

**Table 5:** Correlation between WHO score and other factors in patients with acute leukemia

Parameter	WHO scores	Q1 (overall health)	Q2 (mouth and throat soreness)	Q3‐a (swallowing)	Q3‐b (drinking)	Q3‐c (eating)	Q3‐d (speaking)	Q3‐e (sleeping)	Q4 (overall oral health)
Number of areas WHO scores	**0.858** ** 1.000	0.156 0.040	**0.553** ** **0.521** **	**0.370** * 0.117	**0.370** * 0.117	0.281 **0.250** *	**0.484** ** 0.249	**0.370** * 0.117	**0.534** ** **0.544** **
Q1		1.000	0.156	**0.347** *	**0.347** *	0.276	0.254	**0.365** *	0.235
Q2			1.000	**0.603** **	**0.603** **	**0.617** **	**0.729** **	**0.395** *	**0.863** **
Q3‐a				1.000	**1.000** **	**0.692** **	**0.836** **	**0.676** **	**0.533** **
Q3‐b					1.000	**0.692** **	**0.836** **	**0.676** **	**0.533** **
Q3‐c						1.000	**0.553** **	**0.429** **	**0.659** **
Q3‐d							1.000	**0.559** **	**0.646** **
Q3‐e								1.000	0.283
Q4									1.000

*Note.* Results were obtained via Spearman's correlation analysis; *p* value significance was set at <.05. Significant variables showed in bold text.

Abbreviation: WHO, World Health Organization.

* *p* value <.05.

** *p* value <.01.

## 4 DISCUSSION

A number of hematological malignancies including acute leukemia have manifestations in the oral cavity (Porter, Mercadente, & Fedele, 2018). In a previous retrospective study, the most common oral manifestations of leukemia reported included gingival bleeding (43.2% in AML; 28.6% in ALL), followed by oral ulceration and gingival enlargement (Hou, Huang, & Tsai, 1997). Gingival swelling has always been an early oral sign of an underlying leukemia (Lim & Kim, 2014). For systemic symptoms, patients with acute leukemia frequently have fever (92.2%), followed by fatigue, weakness, and a feeling of helplessness (Hou et al., 1997). Although oral mucositis is one of the commonest side effects when cancer patients experience chemotherapy, there have been few previous studies on CIOM in adult patients with acute leukemia (Dreizen, McCredie, & Keating, 1981; Rimulo, Ferreira, Abreu, Aguirre‐Neto, & Paiva, 2011). This may be because the prevalence of acute leukemia is low, and the disease is rare in adults compared with children. When acute leukemia is left untreated or misdiagnosed, the condition becomes rapidly fatal, within 1 year, in a majority of patients due to severe infection, hemorrhage, gingival, and gastrointestinal bleeding (Estey & Dohner, 2006). Thus, clinicians should be aware of these systemic and oral manifestations of the patients. As far as we know, this is the first study to prospectively investigate the factors that influence the severity of CIOM incidence and symptoms in these patients.

Mucositis is the commonest oral complication among patients receiving chemotherapy (Sonis, 2004). In the present study, the incidence rate was 21.6%, which increased to 50.0% in patients treated with a combination of intensive chemotherapy and HSCT. Oral mucositis, which is directly attributable to antileukemia chemotherapy, has been shown to occur in 20–40% of patients receiving such chemotherapeutic treatment (Figliolia et al., 2008; Scully et al., 2006). Furthermore, mucositis is not only the commonest symptom of HSCT but it is also the most distressing complication, and about 30–50% of patients with HSCT complain that mucositis is the most toxic to them (Bellm, Epstein, Rose‐Ped, Martin, & Fuchs, 2000). In Korea, HSCT procedure was first performed in patients in 1983, and transplants have increased rapidly over the past 35 years (Cho, Lee, & Lee, 2018). The incidence of oral mucositis in patients receiving HSCT has also been reported to be 100% (Vokurka, Steinerova, Karas, & Koza, 2009), whereas the incidence has not yet been investigated in Korean patients who received HSCT. The relationship between HSCT and oral mucositis in adult patients with acute leukemia was first investigated in Korea, and our prospective study could provide the prevalence and clinical characteristics. In our results of linear regression analysis, HSCT certainly increased the severity of CIOM, but not with the subtype of leukemia, age, and gender. Oral mucositis after HSCT can also be a predictor of gastrointestinal toxicity and the onset of hepatic disease (Rapoport et al., 1999; Wingard et al., 1991). To achieve much progress, more studies need to be conducted to alleviate the overall burden and severity of oral mucositis in patients with acute leukemia receiving HSCT.

In general, CIOM starts with the initial injury to cells by chemotherapy either as a direct DNA damage or as an indirect through the action of reactive oxygen species. Chemotherapy may be reflected on directly toxic to the oral cavity, as it performed by killing cells having high‐mitotic activity, such as the cells that make up the oral mucosa (Velten, Zandonade, & Monteiro de Barros Miotto, M. H., 2017). In our study, five out of eight patients were classified WHO Grade 2 with ulceration. A series of antioxidant and detoxifying enzyme and redox‐sensitive transcription factor activations affect the submucosa layer and basal epithelium, leading to tissue damage (Georgiou, Patapatiou, Domoxoudis, Pistevou‐Gompaki, & Papanikolaou, 2012; McKenna, 2000). At its beginning stages or in its mildest form, in contrast, many patients have the more classic and severe form of mucositis that is usually accompanied by ulcerative lesions. Furthermore, development of ulcerative lesions is related with pain and inability to allow ordinary foods (Shankar et al., 2017). In the present study, the severity of CIOM was associated with an increased difficulty in eating. However, this was not a serious enough CIOM‐induced pain to interfere with sleep. This is consistent with reports from previous studies in which the severity of oral mucositis in cancer patients was 40.3% for WHO Grade 1 (Shankar et al., 2017).

In the present study, the most prevalent site of CIOM was the ventral tongue, followed by the buccal mucosa, soft palate, and gingiva in patients with acute leukemia. Additionally, as the number of oral lesions increased, the severity of CIOM also increased. According to Woo et al., majority of ulcers in bone marrow transplant recipients occurred on movable nonkeratinized mucosa are found on buccal mucosa and soft palate (Woo, Sonis, & Sonis, 1990). However, the usual sites of reactivation of intraoral HSV are nonmovable, keratinized mucosae found on the dorsum of the tongue, hard palate, and attached gingiva (Woo et al., 1990). We did not investigate the relationship between HSV reactivation and CIOM, but HSV has been implicated in bone marrow transplant recipients as a crucial etiologic factor in the development of ulcerative mucositis (de Mendonca et al., 2012). Oral manifestations may occur in any of the subtypes of leukemia, but they are known to occur commonly in AML than in ALL (Barrett, 1986). Gingival infiltration of leukemic cells as one of the oral manifestations of AML has been reported in several studies (Fatahzadeh & Krakow, 2008; Lim & Kim, 2014; Wu, Fantasia, & Kaplan, 2002). However, in our study, the CIOM of the gingiva was not present in AML patients and was found in only one case of ALL patients. According to a recent study on oral mucositis in pediatric ALL patients, lip mucosa was the most frequently affected site, with the gingiva being a relatively uncommon site (Ribeiro, Limeira, Dias de Castro, & Ferreti Bonan, 2017). ALL is the most common and the most serious type of childhood cancer, accounting for about 80% of cases of leukemia between 0 and 19 years of age, and the prognosis for ALL is worse in adults over 30 years (Trencsenyi, Bako, Nagy, Kertai, & Banfalvi, 2015). Therefore, only a few prospective studies have analyzed the intraoral findings in adult ALL patients, and further studies are needed to support our results.

This study has some limitations that have to be pointed out. To compare the clinical characteristics of AML and ALL, a relatively small number of participants, especially in ALL, were enrolled. Thus, a detailed and accurate comparison of clinical symptoms between AML and ALL was limited. Additionally, the follow‐up period was limited, and this study does not allow us to draw a concrete conclusion about changes in CIOM over time. However, this study is strong in its prospective nature. It is noteworthy that our results firstly investigated the clinical characteristics of CIOM in adult patients with acute leukemia receiving intensive chemotherapy and/or HSCT. We also investigated how each clinical factor affects the severity of CIOM. These clinical features and relationships were compared between AML and ALL.

CIOM is very debilitating and a painful condition for adult patients with acute leukemia undergoing chemotherapy and a combination therapy with HSCT. CIOM in the buccal mucosa, ventral tongue, and soft palate was associated with an increase in CIOM severity. Impaired eating function was associated with the increased severity of CIOM. Dentists, physicians, and oral health practitioners should be familiar with the oral manifestations as well as the systemic complications of acute leukemia with intensive chemotherapy. With this, we hope that timely management for reducing the CIOM severity and improvement of the patients' quality of life can be provided.

## FUNDING INFORMATION

This research was supported by the Mid‐Career Researcher Program of the National Research Foundation funded by the Ministry of Science & ICT, Republic of Korea (Grant 2017R1A2B4002176) and the Convergence Research Program from the School of Dentistry and College of Medicine, Seoul National University (Grants 860‐20160116 and 800‐20160461).

## CONFLICT OF INTEREST

All authors declare that they have no conflicts of interest.

## CLINICAL SIGNIFICANCE

In this prospective cohort study, we examined the clinical and hematological factors of 37 patients with acute leukemia patients receiving chemotherapy. We also investigated which factors were associated with the severity of oral mucositis in patients with acute leukemia. The severity of oral mucositis was demonstrated with the WHO scoring system.
